# From Fiber to Obstruction: A Case of Small Bowel Obstruction Due to Phytobezoar

**DOI:** 10.1155/carm/9942449

**Published:** 2026-06-13

**Authors:** Sepideh Aarabi

**Affiliations:** ^1^ Department of Emergency Medicine, Sina Hospital, Tehran University of Medical Sciences, Tehran, Iran, tums.ac.ir; ^2^ Research Development Center, Sina Hospital, Tehran University of Medical Sciences, Tehran, Iran, tums.ac.ir

**Keywords:** abdominal pain, phytobezoar, small bowel obstruction

## Abstract

**Introduction:**

Phytobezoars, a rare cause of small bowel obstruction (SBO), account for 0.4%–4% of cases and are most commonly associated with altered gastric motility from prior gastric surgery or systemic conditions like diabetes mellitus and hypothyroidism. Phytobezoars form from indigestible plant fibers that resist enzymatic breakdown, and their incidence may increase in populations with high‐fiber diets. Due to their nonspecific clinical presentation, prompt diagnosis requires a combination of clinical suspicion and imaging studies, particularly computed tomography (CT), by which characteristic intraluminal masses are identified.

**Case Report:**

A case is presented of a 48‐year‐old female with a history of partial gastrectomy for peptic ulcer disease who presented with abdominal pain, nausea, vomiting, and distension. Tenderness and hyperactive bowel sounds consistent with mechanical obstruction were found on examination. A mottled gas‐containing mass in the jejunum, suggestive of a phytobezoar, was identified by CT imaging. Laparotomy was performed, and an enterotomy was carried out to extract the bezoar. Postoperative recovery was uneventful, and dietary counseling was provided to prevent recurrence.

**Conclusion:**

Phytobezoars are an uncommon but important cause of SBO, particularly in patients with risk factors such as prior gastric surgery or high‐fiber diets. Awareness of this condition is crucial for timely diagnosis and management. Surgical intervention remains the mainstay of treatment for complete obstructions, while postoperative dietary counseling and management of underlying conditions are essential to prevent recurrence. The need for heightened clinical awareness to minimize complications associated with phytobezoar‐induced SBO is underscored by this case.

## 1. Introduction

Small bowel obstruction (SBO) accounts for approximately 20% of surgical admissions for acute abdominal conditions. While adhesions, hernias, and malignancies are common causes, bezoars, aggregates of undigested material—are infrequent but significant etiologies. Phytobezoars, composed of undigested plant fibers, are more prevalent in patients with previous gastric surgery, altered gastric motility, or dietary habits predisposing to insufficient fiber digestion. Timely diagnosis and appropriate management are crucial to prevent complications such as bowel ischemia or perforation [1]. Here, a case of SBO secondary to a phytobezoar in a middle‐aged woman is presented, with details of the presentation, diagnosis, and treatment course.

## 2. Case Presentation

A 48‐year‐old woman presented to the emergency department with a 2‐day history of worsening abdominal pain, nausea, vomiting, and abdominal distension. Her bowel movements had ceased 48 h prior to admission. No fever or hematemesis was reported. Her medical history was significant for a subtotal gastrectomy with Billroth II reconstruction performed 15 years ago for peptic ulcer disease. Further dietary history revealed a habit of consuming large amounts of raw vegetables, both of which are known risk factors for phytobezoar formation. Detailed history revealed no psychiatric disorders or cognitive impairment. The patient denied any unusual dietary habits or food cravings. However, she reported consuming a large quantity of persimmons (approximately 5 fruits) 6 days prior to symptom onset during a family gathering. She had not chewed the fruits thoroughly due to partial edentulism. No history of pica, trichotillomania, or other eating disorders was identified. No history of psychiatric illness or abnormal food consumption in the days prior to admission was noted.

On physical examination, the patient appeared distressed. Her vital signs were within normal limits, but abdominal examination revealed marked distension, tympanic percussion, and tenderness in the mid‐abdomen without signs of peritonitis. Bowel sounds were high‐pitched and hyperactive. Laboratory studies were unremarkable except for a mild leukocytosis (12,000/μL).

Multiple air‐fluid levels and dilated small bowel loops, consistent with SBO, were shown on abdominal radiography. On the performed abdominopelvic computed tomography (CT) with IV and oral contrast, a suspicious transitional zone was observed in the distal ileum, accompanied by dilation of the jejunum up to the mid‐ileum, with a maximum diameter of approximately 35 mm. CT imaging demonstrated a markedly dilated small bowel loop in the mid‐ileum (approximately 20 cm proximal to the ileocecal valve), measuring 4.5 cm in diameter, which corresponded to the site of the bezoar impaction. Proximal small bowel loops showed moderate dilatation (up to 3.8 cm), with air‐fluid levels consistent with mechanical obstruction. The transition point was clearly identified at the level of the obstructing mass. An image of a lesion measuring 40 × 25 × 23 mm was identified in the ileal loops located in the right lower quadrant of the abdomen (Figures 1A,B). The distal ileal loops beyond this lesion appeared collapsed. The overall findings were suggestive of an obstruction caused by a bezoar.

**FIGURE 1:**
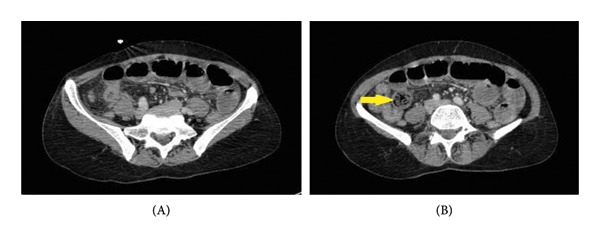
(A, B) In axial images of CT scan a concerning transitional zone was identified in the distal ileum, with associated dilation of the jejunum extending to the mid‐ileum, reaching a maximum diameter of approximately 35 mm. Furthermore, a lesion measuring 40 × 25 × 23 mm was detected within the ileal loops situated in the right lower quadrant of the abdomen (yellow arrow).

The patient’s physical examination revealed tenderness but no signs of peritonitis. Her vital signs were stable. Despite CT findings suggestive of bezoar‐related obstruction, initial conservative management was attempted based on (1) hemodynamic stability with no signs of peritonitis or bowel ischemia, (2) the patient’s strong preference to avoid surgery if possible, (3) literature supporting successful conservative dissolution with Coca‐Cola in selected phytobezoar cases, and (4) the absence of complete obstruction on initial imaging (partial passage of oral contrast noted). However, after 18 h of conservative treatment with nasogastric decompression, bowel rest, and Coca‐Cola lavage, the patient’s symptoms worsened with increasing abdominal distension and pain, prompting surgical intervention. Due to the lack of clinical improvement and persistent obstruction, this conservative approach failed. The patient was then re‐counseled about the necessity of surgical intervention, agreed to the procedure, and subsequently underwent exploratory laparotomy. Due to the lack of clinical improvement and persistent obstruction, exploratory laparotomy was subsequently performed. The decision to proceed with open rather than laparoscopic surgery was based on several factors: (1) significant abdominal distension limiting laparoscopic working space and visualization, (2) the large size of the bezoar (8 cm) identified on CT, requiring a potentially difficult extraction, (3) concern for bowel fragility at the obstruction site after 48 h of impaction, (4) the surgeon’s experience and institutional practice pattern favoring the open approach for established SBO with large bezoars, and (5) the need for thorough exploration to exclude multiple bezoars or underlying pathology given the patient’s prior abdominal surgery. The decision for open surgery over a minimally invasive approach was based on the significant small bowel distension observed on imaging and clinical examination, which is considered a relative contraindication for laparoscopy due to limited working space and increased risk of iatrogenic injury. Intraoperative findings confirmed a phytobezoar causing obstruction in the mid‐jejunum. Enterotomy and removal of the bezoar were performed (Figure 2A,B). Intraoperatively, a hard, ovoid mass measuring approximately 8 × 6 cm was palpated in the mid‐ileum. Following enterotomy, the bezoar was extracted intact. Macroscopic examination revealed a firm, greenish‐brown mass with a rough, fibrous surface texture. Cross‐sectioning demonstrated tightly compacted plant fibers, seeds, and undigested fruit material, consistent with a phytobezoar. The intestinal mucosa at the impaction site showed congestion and mild erosion but no evidence of perforation or ischemia. The remainder of the small bowel was examined and found to be viable. The patient had an uneventful postoperative recovery and was discharged on postoperative day five with dietary counseling to prevent recurrence. The removed bezoar was a firm, oval, greenish‐brown mass composed of compacted vegetable fibers, consistent with a phytobezoar.

**FIGURE 2:**
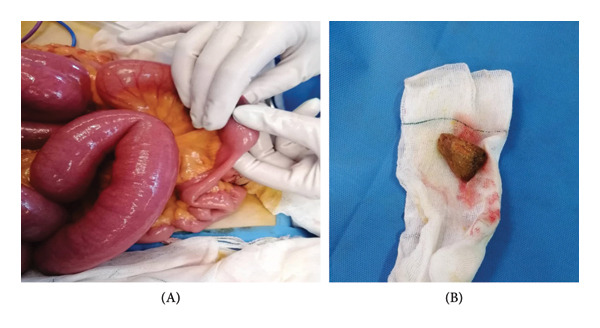
(A, B) An enterotomy was performed, followed by the removal of the bezoar.

## 3. Discussion

Phytobezoars are a rare cause of SBO, accounting for 0.4%–4% of cases. They are formed from the aggregation of indigestible plant fibers (e.g., cellulose, hemicellulose, and lignin) that resist enzymatic breakdown. Risk factors include prior gastric surgery (e.g., partial gastrectomy or Billroth II reconstruction), which leads to delayed gastric emptying and altered motility, as well as inadequate mastication, a high‐fiber diet, and systemic conditions like diabetes mellitus or hypothyroidism that impair gastrointestinal motility. Although rare, the incidence may be higher in regions where high‐fiber diets are common [2].

The novelty of this case lies in several key aspects. First, it provides a detailed temporal link between excessive persimmon (5 fruits) and subsequent phytobezoar formation in a patient with partial edentulism—a modifiable risk factor rarely emphasized in the literature. Second, the case documents the unsuccessful use of Coca‐Cola lavage as a conservative trial before surgery, offering real‐world evidence of its limitations in complete obstruction. Third, the explicit description of the bezoar’s size (8 × 6 cm), macroscopic appearance, and cross‐sectional composition adds valuable pathologic detail often omitted in case reports. Finally, the stepwise clinical decision‐making—including patient preference, failed conservative management, and the rationale for open over laparoscopic surgery—provides a practical framework for clinicians managing similar rare obstructions.

The patient in this case presented with classical symptoms of SBO—abdominal pain, vomiting, distension, and cessation of bowel movements—which, while typical, can lead to delayed diagnosis without high clinical suspicion in at‐risk patients. The diagnosis was confirmed by CT scan, by which a mottled intraluminal mass with a transitional zone was demonstrated, a finding consistent with prior studies. The characteristic mottled gas pattern on CT has a sensitivity of up to 92% for phytobezoars and helps differentiate them from other intraluminal masses such as tumors or fecal matter with high specificity. Similar findings have been reported in other case series [3, 4].

Management depends on the severity and location of the obstruction. In partial obstructions, conservative measures—including nasogastric decompression, intravenous fluid resuscitation, and, in selected cases, endoscopic fragmentation or enzymatic dissolution (e.g., with cellulase or papain)—may be attempted. However, as highlighted in most published series, conservative treatment has a high failure rate (up to 70%), and surgical intervention is often required for complete obstructions or when conservative measures fail. This was evident in the present case, where conservative management was unsuccessful, necessitating laparotomy. Due to significant bowel distension, open surgery was chosen over laparoscopy—a decision supported by other case series that cite marked distension as a relative contraindication to minimally invasive approaches. Intraoperatively, the bezoar was identified as a firm, oval, and greenish‐brown mass composed of compacted vegetable fibers, which is typical of phytobezoars described in the literature [5].

The clinical significance of this case lies in its educational value. The need for clinical suspicion in high‐risk patients is reinforced, and the limitations of conservative treatment, the importance of timely surgical intervention, and the necessity of thorough postoperative dietary counseling are illustrated. Preventive strategies are crucial and include patient education on proper mastication, avoidance of high‐risk foods (e.g., persimmons, celery, and other fibrous vegetables), and management of underlying conditions affecting gastric motility. By presenting this case and reviewing the literature, the importance of early diagnosis, appropriate treatment selection, and preventive measures is emphasized to reduce recurrence and complications associated with phytobezoar‐induced SBO [6].

While laparoscopic management of SBO is increasingly reported, the optimal approach remains controversial. In cases of large phytobezoars with significant bowel distension, open surgery may offer advantages including better tactile feedback, easier extraction of large masses, and more thorough bowel examination. However, it is acknowledged that in experienced hands, laparoscopic or laparoscopic‐assisted approaches could be considered.

## 4. Conclusion

The importance of considering phytobezoars in the differential diagnosis of SBO is highlighted by this case, particularly in patients with risk factors such as prior gastric surgery. Prompt diagnosis and appropriate management can prevent serious complications. Increased awareness and preventive strategies are essential to reduce the burden of this rare condition.

## Author Contributions

The authors have nothing to report.

## Funding

This research received no specific grant from any funding agency in the public, commercial, or not‐for‐profit sectors.

## Ethics Statement

All information, including the photographs and radiological images, has been deidentified. Awareness of the journal’s ethical publication standards is affirmed, and it is confirmed that this case‐based review adheres fully to those guidelines.

## Consent

Written consent was given by the patient for the publication of her clinical information and/or medical images.

## Conflicts of Interest

The author declares no conflicts of interest.

## Data Availability

The data supporting the findings of this case report are included within the article. Raw clinical and imaging data are not publicly available due to patient privacy and ethical restrictions but are available from the corresponding author upon reasonable request and with appropriate institutional approval.
